# Frog-killing chytrid fungus deploys different strategies to regulate intracellular pressure in developmental states that have or lack a cell wall

**DOI:** 10.1016/j.cub.2025.10.013

**Published:** 2025-11-05

**Authors:** Sarah M. Prostak, Katrina B. Velle, Lillian K. Fritz-Laylin

**Affiliations:** 1 Department of Biology, University of Massachusetts Amherst, Amherst, MA 01003, USA; 2 Department of Biology, University of Massachusetts Dartmouth, North Dartmouth, MA 02747, USA; 3 Howard Hughes Medical Institute, University of Massachusetts Amherst, Amherst, MA 01003, USA; 4 Lead contact

## Abstract

Cell morphogenesis is crucial for the physiology of animals and fungi alike. While animals typically shape their cells using the actin cytoskeleton, fungi control cell shape through polarized deposition of new cell-wall material, which is inflated by intracellular osmotic “turgor” pressure. Understanding where and when these mechanisms evolved is essential for understanding cell morphogenesis evolution. To this end, we study chytrid fungi, which have a cell type that lacks a cell wall (the “zoospore”) and a cell type that has a cell wall (the “sporangium”). While chytrid sporangia rely on polarized cell-wall growth to control shape, we previously showed that the “frog-killing” chytrid fungus *Batrachochytrium dendrobatidis* uses actin to control zoospore shape. Whether either zoospores or sporangia also use intracellular pressure regulation in cell-shape control remains an open question. We use live-cell imaging, environmental perturbations, and small-molecule inhibitors to show that *B. dendrobatidis* sporangia generate and maintain turgor pressure, whereas *B. dendrobatidis* zoospores use specialized organelles called contractile vacuoles to pump water out of the cell, thereby keeping internal pressure low. Because chytrid fungi diverged prior to the evolution of the Dikarya—the fungal group including yeasts, mushrooms, and filamentous fungi—these findings suggest that turgor pressure evolved early and that cell morphogenesis underwent a major transition during early fungal evolution. We also suggest that the last common fungal ancestor may have, like chytrid fungi, employed stage-specific strategies for cell-shape control—illustrating how developmental flexibility in cellular mechanisms can serve as a wellspring of evolutionary innovation.

## In brief

Prostak et al. use deep-branching chytrid fungi to investigate the evolution of cell-shape control mechanisms. They find that the chytrid *B. dendrobatidis* alternates between maintaining high turgor pressure in walled sporangia, like other fungi, and reducing pressure in non-walled zoospores via contractile vacuoles, like many amoebae.

## Graphical Abstract

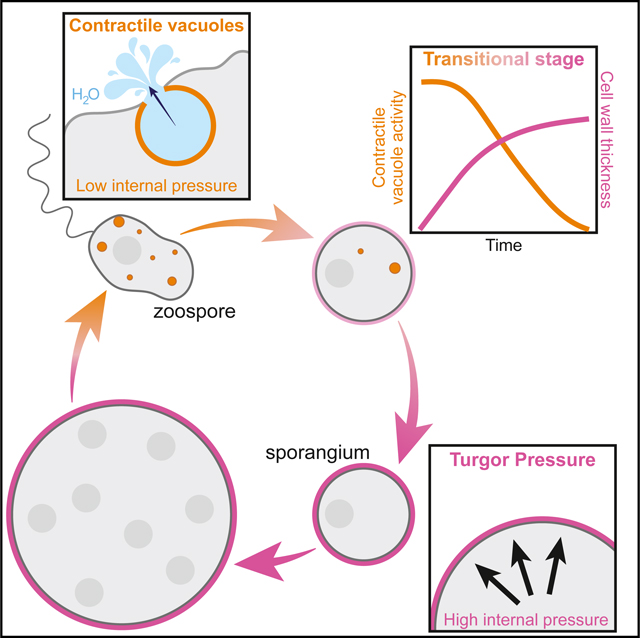

## INTRODUCTION

Control of cell shape is fundamental to organismal form and function, and many cells change shape during development to mediate key aspects of their physiology. Fungal pathogens, for example, actively reshape their cell walls to initiate infection and evade the host immune system. ^1–3^ In turn, white blood cells and unicellular amoebae rely on dynamic shape change to hunt microbes, deforming themselves to crawl across surfaces and engulf their prey. ^4–6^ Other organisms, like chytrid fungi, naturally transition between these states, with one cell type that sculpts their cell walls to facilitate infection and another that uses shape change for locomotion. ^7^ The basis of this functional, developmental, and evolutionary plasticity in the mechanisms controlling cell shape remains unclear.

Fungi and their sister taxa—including animals and various amoebae ^8–10^ —employ distinct mechanisms to control cell shape. Yeasts and filamentous fungi regulate their shape through a combination of directed deposition of cell-wall material and controlled intracellular pressure, known as turgor pressure, which inflates the cell wall. ^11–13^ In contrast, animal cells and amoebae lack cell walls and instead use a dynamic actin cytoskeletal network, called the “actin cortex,” which lies directly under the plasma membrane and provides a scaffold that shapes the cell from within. ^14,15^ Without the external structural support of a cell wall, these cells cannot sustain high internal pressure, as it would push outward on the plasma membrane, causing the cell to burst. Thus, whereas fungi require high internal pressure to maintain cell shape, their sister taxa must maintain low intracellular pressure. This discrepancy suggests that there must have been a major transition in intracellular pressure regulation during fungal evolution. To understand the evolution of cell-shape control, we must therefore understand the evolution of intracellular pressure regulation.

How and when fungal turgor pressure evolved remains largely unexplored, as most research on turgor pressure has focused on Dikarya—the fungal group comprising yeasts, mushrooms, and filamentous fungi ^16^ —all of which are consistently encased in a cell wall. In contrast, fungi that diverged prior to the dikaryotic radiation often alternate between life stages with and without a cell wall. ^7,17^ For example, the “frog-killing” chytrid fungus *Batrachochytrium dendrobatidis* begins life as a unicellular “zoospore” that lacks a cell wall and supports its plasma membrane using actin, much like animal cells and amoebae. ^18,19^ *B. dendrobatidis* zoospores later develop into “sporangia” cells, which build cell walls similar to those of yeast. ^19^ The ability of chytrid fungi like *B. dendrobatidis* to transition between cell types that lack cell walls and cell types with cell walls makes these species valuable models for studying the development of cell-pressure regulation. However, how *B. dendrobatidis* regulates intracellular pressure in either state remains unknown.

Here, we show that *B. dendrobatidis* sporangia use regulated turgor pressure similar to that of Dikarya. In contrast, *B. dendrobatidis* zoospores maintain low intracellular pressure by pumping water out of the cell using specialized organelles called contractile vacuoles, which are commonly found in species of freshwater amoebae. This means that, while cell morphogenesis of sporangia uses cell walls and turgor pressure, zoospore morphogenesis relies on an actin cortex and low intracellular pressure. *B. dendrobatidis*, therefore, naturally transitions between cell-shape control that resembles that used by animal cells and amoebae and cell-shape control similar to that used by dikaryotic cells. These findings suggest that the use of turgor pressure likely evolved before the dikaryotic radiation and that early fungi may have, like chytrid fungi, used distinct cell-shape control strategies at different developmental stages. This developmental plasticity represents a key example of how cellular flexibility can drive evolutionary innovation across the tree of life.

## RESULTS

### *B. dendrobatidis* sporangia have regulated turgor pressure similar to that of Dikarya

**Figure 1.**
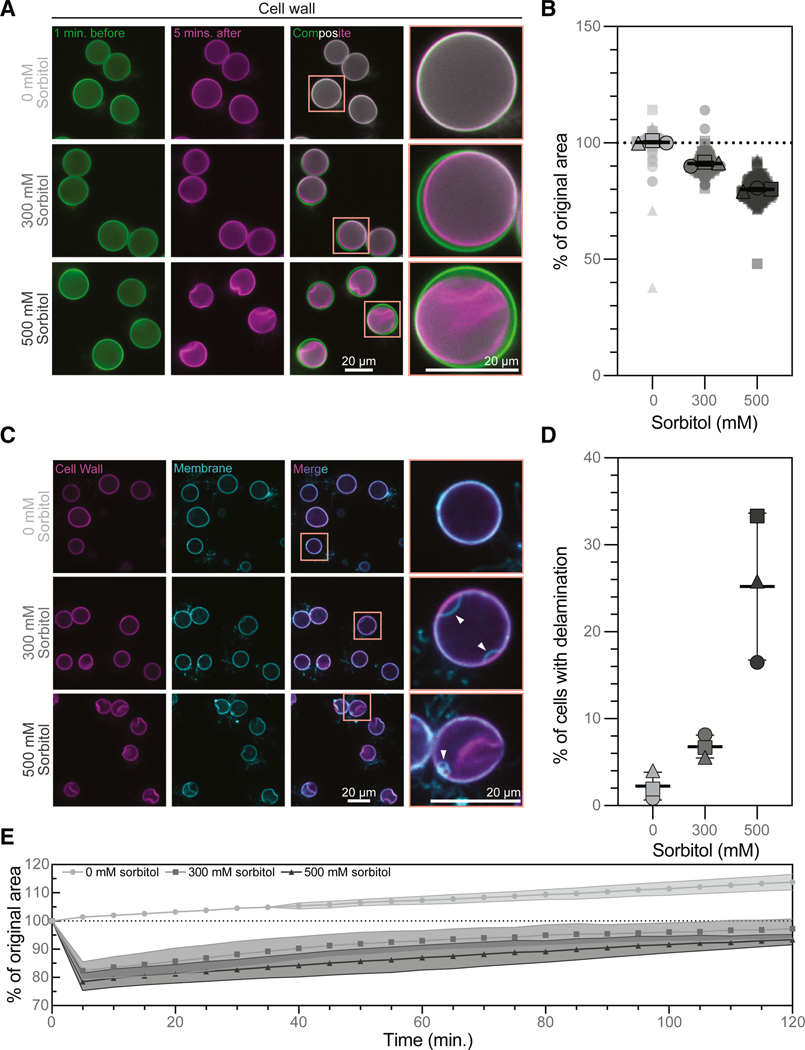
*B. dendrobatidis* sporangia have regulated turgor pressure (A) Representative images of *B. dendrobatidis* sporangia with stained cell walls 1 min before (green) and 5 min after (magenta) treatment with media supplemented with 0, 300, or 500 mM sorbitol. Composite images show the overlay of the time points. All images are adjusted to the same brightness and contrast. (B) Quantification of the change in cellular cross-sectional area after 5 min of the indicated sorbitol treatment, given as the percent of the cell’s cross-sectional area before treatment. Each point represents one cell’s change in cross-sectional area, where the average for each of three independent biological replicates is represented by a shape outlined in black. Mean and standard deviation of the replicate averages are indicated by the black lines. Statistical analysis was performed on the three replicate averages using a one-way ANOVA with Tukey’s multiple comparisons test. For all comparisons: *p* < 0.0001. (C) Representative images of *B. dendrobatidis* sporangia stained for cell walls (magenta) and membranes (cyan) after 5 min of treatment with media supplemented with 0, 300, or 500 mM sorbitol. Merged images show the overlay of membrane and cell wall. White arrowheads indicate points at which the membrane is delaminated from the cell wall. All images for each stain are adjusted to the same brightness and contrast. (D) Quantification of the percent of cells with clear delamination of the membrane from the cell wall in the focal plane at 5 min post treatment with the indicated sorbitol treatment. Mean and standard deviation of three independent biological replicates (black-outlined shapes) are indicated by black lines. Statistical analysis was performed on the average percentages using a one-way ANOVA with Tukey’s multiple comparisons test: 0 vs. 300 n.s.; 0 vs. 500 *p* = 0.0033; 300 vs. 500 *p* = 0.0099. (E) Quantification of cellular cross-sectional area over 120 min of the indicated sorbitol treatment, given as the percent of the cell’s area before treatment. See also Figures S1 and S2A, and Videos S1 and S2, and Data S1.

To understand the evolution of cell-shape control in the fungal kingdom, we must understand the development of turgor pressure. Because *B. dendrobatidis* sporangia are encased by cell walls, like the cells of dikaryotic fungi, we hypothesized they too maintain regulated turgor pressure. Turgor pressure is an osmotic system and its presence is typically demonstrated by cell shrinkage and recovery upon hyperosmotic shock induced by the non-metabolizable sugar sorbitol. ^16,20,21^ We therefore stained the cell walls of *B. dendrobatidis* sporangia using Evan’s Blue, then imaged cell cross-sections before and after sorbitol treatment and observed concentration-dependent cell shrinkage (Figure 1A). While control cells treated with media alone maintained a constant cross-sectional area (100% ± 0.61% of their original cross-sectional area, Figure 1B), sporangia treated with media supplemented with 300 mM sorbitol for 5 min shrank to 91% ± 0.91% of their original cross-sectional area (Figure 1B; *p* < 0.0001), and sporangia in 500 mM sorbitol shrank to 79.94% ± 0.75% of their original cross-sectional area (Figure 1B; *p* < 0.0001). These data represent a cross-sectional area change of approximately 10% upon 300 mM sorbitol treatment and 20% upon 500 mM sorbitol treatment. To confirm that quantifying single cross-sections is a reasonable proxy for changes in volume, we measured the volume of a subset of cells (*n* = 50) using 3D imaging and thresholding as well as estimated their volume using the cross-sectional area, then determined the before and after treatment ratio with both of these metrics. The change in cell size 5 min after sorbitol treatment calculated from both the measured and estimated volumes agree within 10% (Figure S1C), validating that cross-sectional area can be used to assess changes in cell size in *B. dendrobatidis* sporangia. Because largescale cell shrinkage during hyperosmotic shock has been associated with plasma membrane delamination from the cell wall in plants and other fungi,^20,22,23^ we next checked whether something similar may occur in *B. dendrobatidis* sporangia. We stained membranes with FM4–64, the cell wall with calcofluor white, and again imaged sporangia cross-sections after 5 min of sorbitol treatment (Figure 1C; Video S1). While only 2.26% ± 1.60% of control sporangia and 6.8% ± 1.31% of sporangia treated with 300 mM sorbitol exhibited plasma membrane delamination (Figure 1D; not significant; see also Figure S1D), 25.20% ± 8.45% of cells treated with 500 mM sorbitol showed clear delaminations (Figure 1D; *p* = 0.0033, see also Figure S1D). Taken together, these results indicate that chytrid sporangia are, like dikaryotic fungi, under turgor pressure.

Dikaryotic fungi not only possess turgor pressure but also actively regulate it in response to environmental changes. ^16^ Budding yeast, for example, recover to approximately 90% of their pre-stress volume within 1 to 2 h of hyperosmotic shock. ^24,25^ To determine whether *B. dendrobatidis* also actively regulates its turgor pressure, we therefore evaluated its ability to recover volume during long-term exposure to hyperosmotic conditions. To do this, we measured the area of cross-sections through *B. dendrobatidis* sporangia every 5 min during 2 h of sorbitol treatment. While control cells steadily increased in area over the 2 h imaging period—consistent with normal growth—cells treated with 300 mM sorbitol shrank after 5 min to 82.3% ± 3.7% of their original area, and after 2 h the cells returned to 98.3% ± 3.8% of their original area (Figure 1E; Video S2). Cells treated with 500 mM sorbitol shrank after 5 min to 78.3% ± 3.3% of their original area, and after 2 h they returned to 93.7% ± 2.1% of their original area (Figure 1E; Video S2). This represents an area increase of approximately 16% over the time of recovery for both 300 and 500 mM sorbitol treated cells. These findings indicate that *B. dendrobatidis* sporangia actively control their turgor pressure with dynamics similar to those of Dikarya.

Dikarya regulate turgor pressure primarily by controlling internal osmolyte concentrations using the high-osmolarity glycerol (HOG) signaling pathway. ^16^ The HOG pathway has three main classes of proteins: (1) osmo-sensors that detect changes in osmolarity via cell-wall integrity, molecular crowding, and other mechanisms ^16,21,26^, (2) a mitogen-activated kinase (MAPK) cascade, ending with the MAPK Hog1, which modulates gene expression ^27–29^, and (3) signal transducers that connect the osmosensors and MAPKs. To determine whether *B. dendrobatidis* and other chytrids may use the HOG pathway to regulate turgor pressure, we performed a genomic survey to identify putative homologs of major HOG pathway genes in the genomes of three species representing several chytrid phyla—*B. dendrobatidis*, *Spizellomyces punctatus*, and *Allomyces macrogynus*—along with a variety of dikaryotic fungi. We found that osmosensor distribution varies across chytrid species, with some having multiple putative osmosensors and others lacking any clear putative homolog (Figure S2A). Furthermore, *B. dendrobatidis* and *A. macrogynus* have putative homologs for the entire HOG MAPK cascade, whereas *S. punctatus* appears to be missing the mitogen-activated kinase kinase kinases Ssk2/22 and Ste11 (Figure S2A). Finally, there is large variation in the repertoire of transducers among chytrid species, with *B. dendrobatidis* having only two putative homologs of the eight investigated proteins, while *A. macrogynus* has five and *S. punctatus* has six (Figure S2A). Taken together, these results suggest that *B. dendrobatidis* and other chytrids may use a version of the HOG pathway to actively regulate turgor pressure.

### *B. dendrobatidis* zoospores have contractile vacuoles that respond to extracellular osmolarity

Having determined that *B. dendrobatidis* sporangia actively maintain turgor pressure, we wondered how the zoospores that lack cell walls regulate their intracellular pressure. Because of their extreme osmotic environment, freshwater organisms that lack cell walls have evolved a variety of mechanisms to regulate internal pressure. Freshwater ciliates and amoebae, for example, use membrane-bound contractile vacuoles that literally pump water out of the cell, using ion gradients to pull water into the vacuole lumen from the cytoplasm and cytoplasmic pressure to expel the water through pores connected to the extracellular environment. ^30–35^ This activity can be visualized by a cycle of slow contractile vacuole growth followed by a rapid disappearance. Because contractile vacuoles have been documented in species from across the eukaryotic tree, ^36–41^ we hypothesized that *B. dendrobatidis* zoospores may use a similar mechanism to avoid bursting in their freshwater habitats. Supporting this idea, structures resembling contractile vacuoles have been observed by light and electron microscopy in the zoospores of several chytrid species, ^42–45^ with the presence of these vacuoles correlated with the osmolarity of the liquid used for zoospore collection.

**Figure 2.**
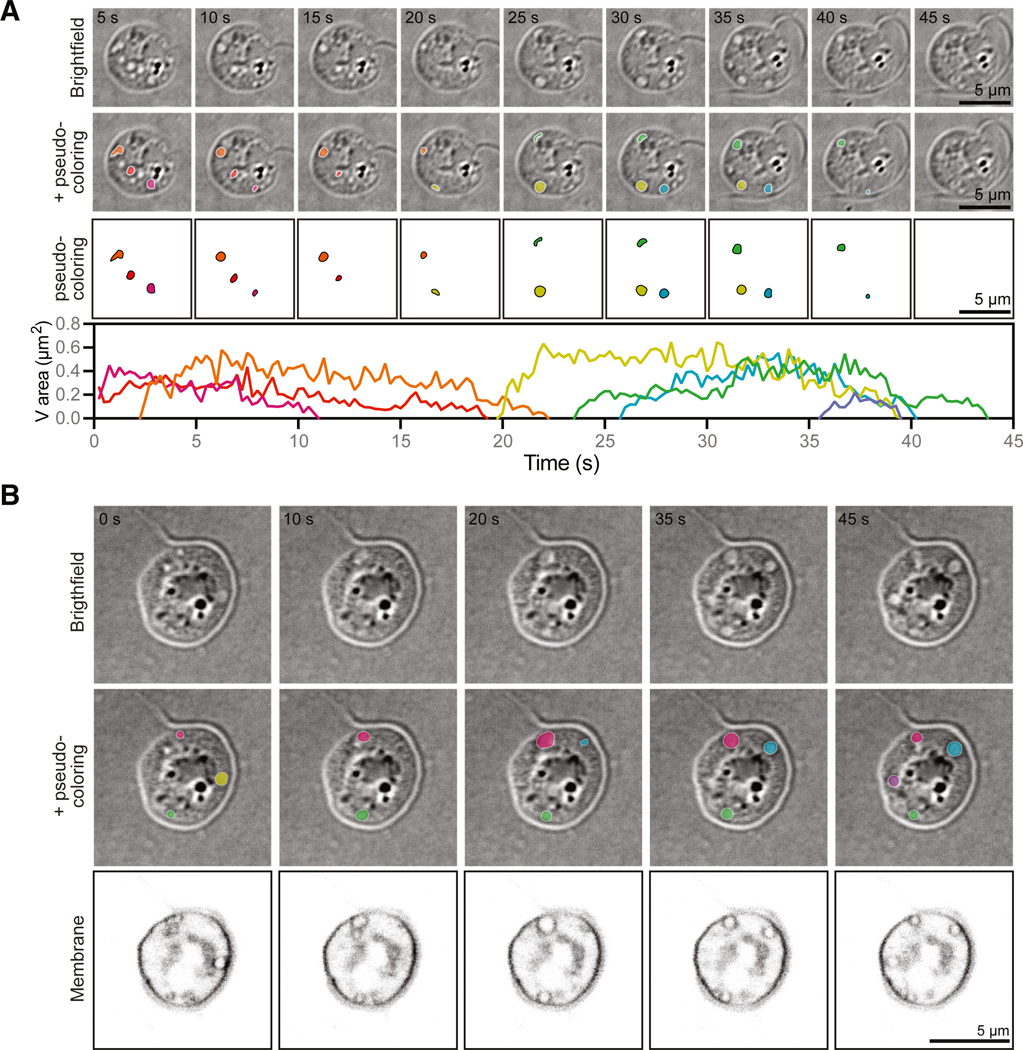
*B. dendrobatidis* zoospores have small, circular, membrane-bound organelles that grow and shrink over time (A) Representative time-lapse images of *B. dendrobatidis* zoospores under agarose through multiple growth and shrinkage events of small, circular organelles (top). These organelles were tracked as they grew and shrank and are pseudo-colored by organelle (middle). The area of each organelle is graphed over time (bottom). (B) Representative time-lapse images of membrane-stained *B. dendrobatidis* zoospores under agarose (bottom), with the small organelles observed in bright-field (top) pseudo-colored by organelle (middle). All membrane images are adjusted to the same brightness and contrast. All bright-field images are on an inverted look-up table. See also Figures S1B and S2B and Video S3 and Data S1.

To explore whether chytrid zoospores use contractile vacuoles, we searched for organelles consistent with the defining features of these structures: membrane-bound compartments whose number, size, and/or disappearance rate vary in response to external osmolarity. ^36,39,42,46^ Because contractile vacuole location is highly variable, and because *B. dendrobatidis* zoospores swim rapidly out of sight, we imaged zoospores under an agarose pad to trap and flatten the entire cell volume into more or less the same focal plane. Although confinement may alter the absolute dynamics of these structures, this approach is necessary for their visualization and also allows us to use contractile vacuole area as a proxy for its volume. ^36^ Live-cell imaging of *B. dendrobatidis* zoospores under agarose using bright-field microscopy revealed small circular structures that grow and shrink largely asynchronously over time (Figure 2A; Video S3). To determine whether these structures are membrane-bound organelles, we stained *B. dendrobatidis* zoospores using the membrane dye FM4–64, imaged them using scanning point confocal fluorescence microscopy, and observed that these dynamic structures are indeed enclosed by membranes (Figure 2B; Video S3). Taken together, these results show that *B. dendrobatidis* has membrane-bound organelles that, like contractile vacuoles in other organisms, grow over time and then rapidly disappear.

**Figure 3.**
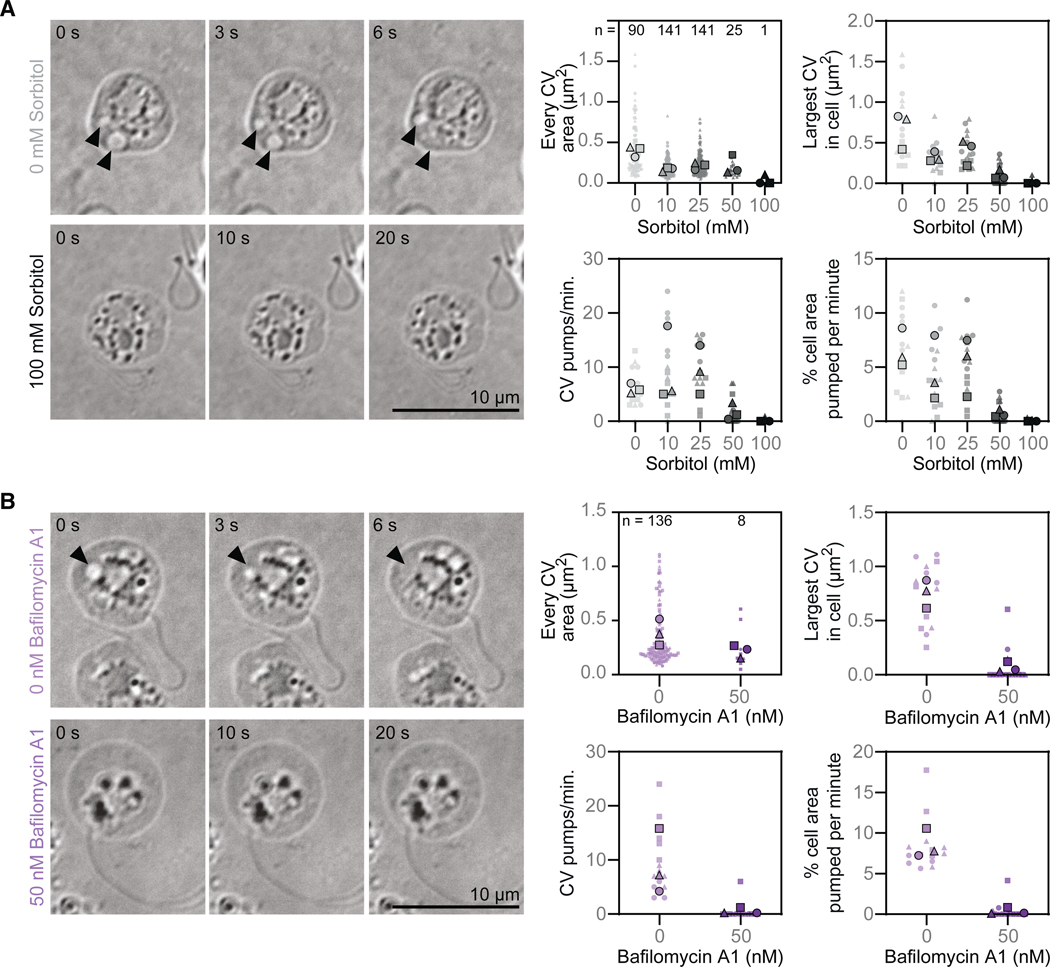
Small, membrane-bound organelles in *B. dendrobatidis* zoospores respond to hyperosmotic shock and rely on vacuolar-ATPase function (A) Representative time-lapse images of *B. dendrobatidis* zoospores under agarose in the given sorbitol concentrations (left) and quantification of the area, pumping rate, and percent of cell area pumped per min of all of the putative contractile vacuoles (CVs) from five random cells per sorbitol treatment (right). Black arrowheads indicate growing and shrinking organelles. Number of growing and shrinking organelles quantified indicated by *n*. Black outlined shapes indicate the mean of each of three biological replicates. (B) Representative time-lapse images of *B. dendrobatidis* zoospores under agarose, treated with or without 50 nM of the vacuolar-ATPase inhibitor Bafilomycin-A1 for 35 min (left), and quantification of vacuole area, pumping rate, and percent of cell area pumped per min for all contractile vacuoles (CVs) in five random cells per treatment (right). Black arrowheads indicate growing and shrinking organelles. Number of growing and shrinking organelles quantified indicated by *n*. Black outlined shapes indicate the mean of each of three biological replicates. All bright-field images are on an inverted look-up table. See also Figure S3 and Video S4.

As osmoregulators, contractile vacuoles are defined by changing volume and/or pumping rate in response to changes in environmental osmolarity. In the lab, this is typically measured by observing changes in contractile vacuole activity in the presence of varying concentrations of sorbitol. ^36,47^ We therefore imaged zoospores under a range of sorbitol concentrations and quantified the number, size, and dynamics of the membrane-bound organelles. We found that, as sorbitol concentration increases, the average area of the largest dynamic organelle per cell decreases, starting from an average of 0.68 ± 0.22 μm^2^ in control cells treated with buffer alone to as small as 0 ± 0.01 μm^2^ in cells treated with 100 mM sorbitol (Figure 3A; Video S4). The frequency of disappearance of these organelles also responded to extracellular osmolarity. The organelles in control cells averaged 6 ± 0.92 disappearance events per min, and this rate generally decreased as sorbitol concentration increased, with the organelles of cells treated with 100 mM sorbitol averaging 0.07 ± 0.12 disappearance events per minute (Figure 3A). Given the cell-to-cell variability of largest vacuole size and disappearance rate, both within and between biological replicates, we quantified the vacuoles’ percent of the total cell area and found, again—and with slightly lower variability—that the percent of the total cell area occupied generally decreased with increasing sorbitol concentrations (Figure 3A). These results show that the dynamic organelles observed in *B. dendrobatidis* respond to changes in external osmolarity—indicating that the disappearance of the vacuoles represents pumping used to regulate internal pressure—and therefore fit the definition of contractile vacuoles.

Contractile vacuole function in other species depends on vacuolar-ATPases (v-ATPases), which create an ion gradient to facilitate water flow into the contractile vacuole through aquaporins. ^30–35^ To explore whether chytrids may use the same molecular mechanisms to power vacuole filling, we first searched for v-ATPases and aquaporins in the genomes of three chytrid species representing different chytrid phyla and found putative homologs of all subunits of the v-ATPase complex, ^48,49^ along with several aquaporins (Figure S2B). To test whether v-ATPase activity is important for contractile vacuole function in *B. dendrobatidis*, we treated zoospores with Bafilomycin-A1, a v-ATPase inhibitor that disrupts contractile vacuole function by preventing contractile vacuole refilling. (The predicted *B. dendrobatidis* v-ATPase subunits have all the conserved residues for Bafilomycin-A1 binding and function, ^50^ Figure S3A). Treating *B. dendrobatidis* zoospores with 50 nM Bafilomycin-A1 for 35 min drastically reduced contractile vacuole size: the area of the largest contractile vacuole in Bafilomycin-A1-treated cells was 0.07 ± 0.05 μm^2^ compared with 0.75 ± 0.13 μm^2^ in cells treated with buffer alone (Figure 3B; Video S4). We also found that Bafilomycin-A1 treatment had drastic effects on contractile vacuole pumping, with the contractile vacuoles of Bafilomycin-A1-treated cells pumping an average of 0.53 ± 0.57 pumps per min compared with 9.07 ± 6.02 pumps per min in control cells (Figure 3B). Normalizing for cell size, we calculated that Bafilomycin-A1-treated cells pump only 0.35% ± 0.41% of their area per min compared with control cells, which pump 8.53% ± 1.79% of their area per min (Figure 3B). Because v-ATPase activity is required for contractile vacuole refilling, we next quantified the number of *B. dendrobatidis* zoospores that undergo multiple pumping cycles in the presence of this drug. Of cells with active contractile vacuoles at the beginning of imaging, 95.5% ± 2.3% of control cells underwent multiple pump cycles compared with only 15.2% ± 11.5% of Bafilomycin-A1-treated cells (Figure S3C, *p* = 0.0007). Finally, because zoospores with nonfunctional contractile vacuoles are likely to die due to being overwatered, we quantified the percentage of cells that died over the 3-min imaging period. We found that only 0.2% ± 0.21% of control cells died, while 2.42% ± 0.36% of Bafilomycin-A1-treated cells died (Figure S3D, *p* = 0.0008). To confirm that the results we see are due specifically to v-ATPase inhibition, we fixed and stained cells treated with Bafilomycin-A1 and saw no aberrations in their actin or nuclei, indicating that there are no obvious off-target effects of this drug (Figure S3B). We also treated cells with another v-ATPase inhibitor, Concanamycin-A, measured contractile vacuole dynamics, and found similar results to Bafilomycin-A1 treatment (Figures S3E and S3F). Taken together, these results suggest that the contractile vacuoles of *B. dendrobatidis* rely heavily on v-ATPase activity and are critical to survival in freshwater.

### *B. dendrobatidis* contractile vacuoles slow their pumping during cell-wall accumulation

**Figure 4.**
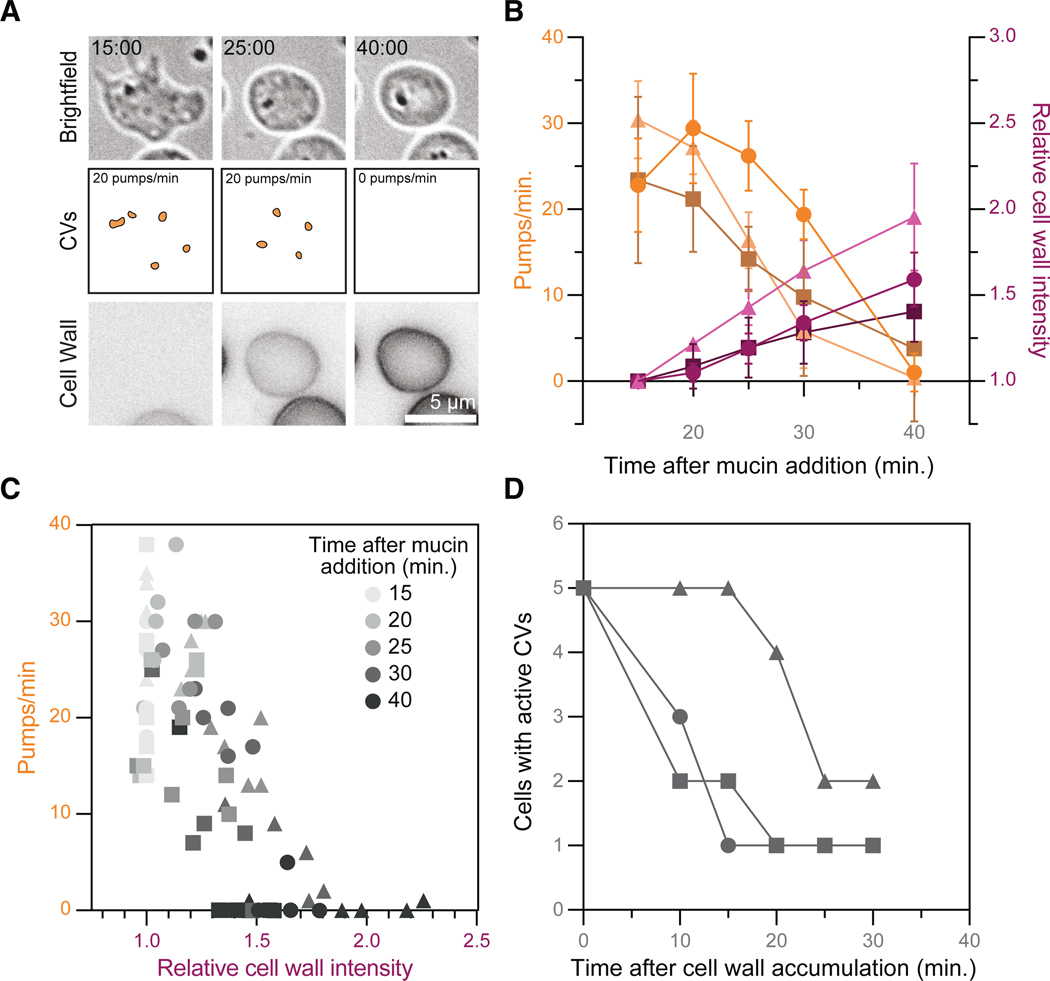
The pumping of *B. dendrobatidis* zoospores’ contractile vacuoles slows during cell-wall assembly (A) Representative time-lapse images of cell-wall-stained, confined *B. dendrobatidis* zoospores after mucin treatment to induce encystation. Contractile vacuoles are highlighted (middle). Bright-field images are on an inverted look-up table. All cell-wall images are adjusted to the same brightness and contrast. (B) Quantification of the contractile vacuole pumping rate (shades of orange, left axis) and the relative cell-wall intensity (shades of magenta, right axis) over time after mucin treatment. Mean and standard deviation values for each time point for three independent replicates (shapes) are shown. Some error bars are not shown, as they were smaller than the shape itself. (C) All cells quantified in (B), graphed by pumping rate vs. relative cell-wall intensity. Time after mucin treatment is indicated in shades of gray for each shape. Each shape represents data from an independent replicate. (D) Quantification of the number of cells with active contractile vacuoles (>0 pumps/min) over time after cell-wall accumulation. Cell wall was considered to have accumulated at the first time point where total cell-wall intensity was at least 20% greater than background. Each shape represents an independent replicate.

Having determined that *B. dendrobatidis* cells that lack cell walls reduce intracellular pressure, whereas *B. dendrobatidis* cells with cell walls boost it, we next wondered what happens when *B. dendrobatidis* transitions between these two states. Because maintaining high intracellular pressure is incompatible with a wall-less cell type, we hypothesized that this transition must involve a gradual shift from contractile vacuole activity to turgor-based pressure regulation, coordinated by regulatory mechanisms that link pressure control to cell-wall assembly. To test this hypothesis, we visualized contractile vacuole activity and cell-wall accumulation in cells undergoing the zoospore-to-sporangium transition—a developmental process called encystation—which can be rapidly triggered by treatment with purified mucin. ^51^ We treated cells with mucin, immediately flattened them, and imaged contractile vacuole activity and cell-wall accumulation after 15, 20, 25, 30, and 40 min (Figure 4A). We found that contractile vacuoles pump on average 25.53 ± 7.34 pumps per min at 15 min after mucin treatment, and this rate decreases to about 1.73 ± 4.95 pumps per min at 40 min after treatment (Figure 4B). During these 25 min, the total cell-wall intensity increased by 1.65 ± 0.32 times (Figure 4B). To visualize this relationship directly, we plotted pumping rate against cell-wall intensity and found a clear trend: as cell-wall intensity increases, contractile vacuole pumping rate decreases (Figure 4C). We next wondered how long it takes after appreciable cell-wall accumulation for contractile vacuoles to stop pumping. To this end, we identified the first time point at which the total cell-wall intensity of an individual cell was at least 20% higher than the background, determined when that cell’s pumping rate dropped to zero, and then calculated the time difference between these two events. Although the timing varied across cells, most stopped pumping within 10–25 min of appreciable cell-wall accumulation (Figure 4D). This delay suggests that the switch from contractile vacuole activity to turgor-based pressure regulation happens gradually, likely to allow for sufficient cell-wall thickness to counter the force of turgor pressure on the membrane, and/or for the cell to produce sufficient osmolyte to modulate water influx. Either way, these two scenarios imply the existence of molecular mechanisms allowing cross-talk between cell-wall assembly, turgor pressure regulation, and/or contractile vacuole activity.

## DISCUSSION

Here, we show that different *B. dendrobatidis* cell types use distinct modes of intracellular pressure regulation. While sporangia that have cell walls actively maintain high turgor pressure, zoospores that lack cell walls use contractile vacuoles to maintain low intracellular pressure. Together with previous work showing that *B. dendrobatidis* zoospores migrate using a dynamic actin cortex, ^18^ our findings suggest that chytrids naturally alternate between two fundamentally different modes of cell-shape control: the use of an actin cortex similar to those of animals and amoebae in the wall-less zoospore and a canonical fungal system in the walled sporangium. This duality makes *B. dendrobatidis* a valuable model for studying the evolution and development of intracellular pressure regulation and cell morphogenesis.

Because chytrid fungi diverged before the dikaryotic radiation, ^52^ our discovery of regulated turgor pressure in *B. dendrobatidis* sporangia suggests that this mechanism of pressure control arose very early in fungal evolution and may have been present in the last common ancestor of fungi. The importance of turgor pressure for morphogenesis in Dikarya ^11,12,53^ makes chytrids an important point of comparison for understanding the evolution of multicellular fungi. Importantly, the ability of *B. dendrobatidis* to toggle between shape control strategies associated with either animals or fungi suggests that the last common ancestor of fungi may have deployed similar context-dependent mechanisms. This finding implies that, rather than a linear evolutionary shift from amoeboid to turgor-based morphogenesis, the evolution of fungal cell-shape control may have involved the co-option and reconfiguration of pre-existing systems.

In addition to shedding light on the evolution of cell-shape control in fungi, this work advances our understanding of *B. dendrobatidis* pathogenesis. As an amphibian pathogen, *B. dendrobatidis* encounters dramatically different osmotic environments over the course of its life cycle. Zoospores swim in freshwater, where the near-zero osmolarity creates a constant influx of water that must be actively managed. In contrast, sporangia develop within frog epithelial tissues, ^54^ where the osmolarity is approximately 200–300 mOsM, ^55^ resulting in a far lower osmotic gradient and reduced water influx. The ability to regulate intracellular pressure across these conditions is likely essential for *B. dendrobatidis*’s survival and infection success. Moreover, like other fungal pathogens, ^1,56^ *B. dendrobatidis* sporangia may use turgor pressure during its invasion of host cells or tissues. ^54^

Finally, these results may provide clues about the environmental context in which fungi evolved. Although hotly debated, the two dominant hypotheses propose that fungi originated in either marine or freshwater habitats.^57^ Our finding that *B. dendrobatidis* zoospores use contractile vacuoles—organelles typically associated with freshwater organisms—is consistent with the idea that early fungi evolved in aquatic environments, where regulation of intracellular pressure would have been crucial for survival. The ability of early fungi to transition between using contractile vacuoles that facilitate survival in freshwater environments and using turgor pressure/cell-wall systems that enable survival in terrestrial environments may have been crucial for the conquest of land. This flexibility in cellular morphogenesis exemplifies how developmental plasticity in core cellular mechanisms can facilitate organisms’ colonization of diverse ecological niches. This principle extends beyond the fungal kingdom, as developmental flexibility in cellular mechanisms serves as a wellspring of evolutionary innovation across the tree of life.

## RESOURCE AVAILABILITY

### Lead contact

Requests for further information and resources should be directed to and will be fulfilled by the lead contact, Lillian Fritz-Laylin (lfritzlaylin@umass.edu).

### Materials availability

This study did not generate new unique reagents.

### Data and code availability

- All data reported in this paper will be shared by the lead contact upon request.
- All original code and analysis pipelines have been deposited at Zenodo and are publicly available at https://doi.org/10.5281/zenodo.16329705 as of the date of publication.
- Any additional information required to reanalyze the data reported in this paper is available from the lead contact upon request.

## STAR★METHODS

### EXPERIMENTAL MODEL AND SUBJECT DETAILS

#### Turgor pressure experiments

*Batrachochytrium dendrobatidis* JEL423 (*Bd*) zoospores from a culture grown at 24° C in 1% tryptone (w/v; Sigma # T7293) were harvested by filtering through a sterile 40 μm filter (CellTreat #229481), then again through a whatman grade 1 filter paper (Cytiva #1001–325) syringe filter (Fisher Scientific #NC9972954). Filtered cells were counted, then diluted to 2×10^5^ cells/mL into fresh 1% tryptone and seeded into a 12-well glass-like polymer bottom plate (Cellvis #P12–1.5P). The plate was sealed with parafilm and incubated at 24° C for 48 hours.

#### Contractile vacuole, transition experiments

*Bd* zoospores were grown, harvested, and filtered as for turgor pressure experiments. Filtered cells were centrifuged at 2500 rcf for five minutes, washed twice in 0.1X Bonner’s salts (1X Bonner’s salts ^78^: 10 mM sodium chloride, 10 mM potassium chloride, 2.7 mM calcium chloride), and resuspended in 2 mL 0.1X Bonner’s salts.

### METHOD DETAILS

All experiments were performed at room temperature and were performed on three independent populations of sporangia or zoospores harvested on different days (biological replicates). Sample sizes were not predetermined by statistical methods.

#### Hyperosmotic shock of sporangia

##### Short-term sorbitol treatments

Cells were stained for the cell wall using 0.1% calcofluor white with an Evans Blue counterstain (v/v; Sigma #18909) in 1% tryptone. After one minute of imaging, the media in the well was removed and replaced with either fresh 1% tryptone or 1% tryptone supplemented with 300 mM or 500 mM sorbitol (Sigma #S1876). The time of media replacement with respect to the start of the imaging acquisition was recorded. This experiment was also repeated once more, but imaged using 3D imaging to assess the difference between cell size change based on cross-sectional area compared to measured volume.

##### Membrane and cell wall staining

Cells were incubated with 0.1% calcofluor white without any counterstain (v/v; Sigma #910090) for 20 minutes in 1% tryptone to stain the cell wall. Cells were washed three times with 1% tryptone and then stained for the cell membrane using 10 μM FM4–64 (Invitrogen #T13320). Sorbitol treatments were performed as in the short term experiments, except the treatment solution was supplemented with 10 μM FM4–64.

##### Long term sorbitol treatments

Cells were stained as in the short term experiments. After the first time lapse frame for each experimental condition was taken, fresh 1% tryptone or 1% tryptone supplemented with sorbitol was added to the appropriate wells to final concentration of 300 or 500 mM sorbitol. The second time lapse frame was considered to be the five minute post-treatment time.

#### Viewing contractile vacuoles in zoospores

##### Sorbitol treatments under agarose

1.5% low-melt agarose (w/v; Thermo Scientific #R0801) pads were prepared using 0.1X Bonner’s supplemented with 0, 10, 25, 50, or 100 mM sorbitol. Concentrated cells prepared as described above (see “Experimental model and subject details”) were added to the middle of a well of a 12-well glass-bottom plate (Cellvis #P12–1.5H-N). An agarose pad was gently placed over the cells and left for five minutes before excess liquid was removed from the edges of the pad via pipetting. To allow acclimation to the osmotic environment and adequate confinement, the cells were left for 30 minutes, during which any liquid around the edge of the pad was occasionally removed using the corner of a kim wipe. After this time, cells were imaged.

##### Membrane staining under agarose

1.5% low-melt agarose (w/v) pads supplemented with 10 μM FM4–64 were prepared using 0.1X Bonner’s salts. Concentrated cells were added to the center of a 12-well glass-bottom plate and FM4–64 was added to a final concentration of 10 μM. Then, the agarose pad was gently placed over the cells. After five minutes, excess liquid was removed via pipetting. After five more minutes, the remaining liquid around the edge of the agarose pad was removed with the corner of a kim wipe, and cells were imaged.

##### Chemical inhibitors under agarose

1.5% low-melt agarose (w/v) pads with either 0 or 50 nM Bafilomycin-A1 (Cayman Chemical #11038, resuspended in methanol) were prepared using 0.1X Bonner’s. Concentrated cells were added to the middle of a well of a 12-well glass-bottom plate and propidium iodide (PI, Invitrogen #P3566) was added to a final concentration of 0.1% (v/v). For treated cells, Bafilomycin-A1 was added to the well to a final concentration of 50 nM. The appropriate agarose pads were added over the cells and liquid was removed as described for the sorbitol treatments under agarose and cells were imaged. These experiments were repeated using 0.2 and 1 μM Concanamycin-A (Tocris #2656), and appropriate methanol vehicle and negative controls.

##### Chemical inhibitors, actin, and nuclear staining

To test for potential off-target effects of Bafilomycin-A1 (Figure S3B), concentrated cells were added to a Concanavalin-A-coated (Sigma #C2010) well of a 96-well glass bottom plate (Azenta #MGB069–1-2-LG-L) to adhere cells to the glass. Bafilomycin-A1, or equivalent volume of 0.1X Bonner’s or methanol, was added to a final concentration of 50 nM and incubated for 35 minutes at room temperature. Cells were washed thrice with 0.1X Bonner’s before being fixed with 4% paraformaldehyde in 50 mM sodium cacodylate (pH = 7.3) on ice for 20 minutes. Cells were washed thrice with PEM buffer (100 mM PIPES pH 6.9, 1 mM EGTA, 0.1 mM magnesium sulfate) before being permeabilized with 0.1% Triton-X 100 (Sigma #T9284), and stained for DNA using 1:2000 DAPI (0.5 mg/mL, Life Technologies #D1306) in PEM for 10 minutes at room temperature. Cells were again washed thrice with PEM before being stained for actin using 1:1000 AlexaFlour488 phalloidin (∼66 μM, Cell Signaling Technology #8878S) for 30 minutes at room temperature. Phalloidin was washed out and cells imaged in PEM.

##### Encystation

Concentrated cells prepared as described above (see “Experimental model and subject details”) were added to the middle of a well of a 6-well glass bottom plate (Cellvis #P06–1.5H-N) with calcofluor white with Evans Blue (Sigma #18909) at a final concentration of 0.1% (v/v). The solution was mixed gently before the addition of mucin (Sigma #M1778) to a final concentration of 10 mg/mL. Cells were then immediately confined using a dynamic cell confiner (4Dcell) with a PDMS suction cup and 1 μm pillar-height coverslip presoaked in 0.1X Bonners and 0.1% (v/v) calcofluor white solution. The coverslip was gently lowered onto the cells to prevent bursting and cells imaged after 15, 20, 25, 30, and 40 minutes of mucin treatment.

##### Microscopy

All imaging was performed on one of three inverted microscopes (Ti2-Eclipse; Nikon) at room temperature. Zoospore imaging used a 1.5X tube lens in addition to the given objective described below. Microscope configurations are as follows. Microscope 1 was equipped with a Prime BSI Express camera (Teledyne). Brightfield illumination was supplied by a multi-wavelength LED lightsource (CoolLED pE-300 white). For widefield fluorescence imaging, illumination was supplied by the included diodes in the LED lightsource at 5% power, and combined with a multi-bandpass filter set (Chroma 89404), with the following excitation wavelengths and emission filters: Cy5, 550 nm, Chroma ET697/60m; TRITC, 550 nm, Chroma ET595/33m; GFP, 460 nm, Chroma ET519/26m; DAPI, 400 nm, Chroma ET434/32m. Microscope 2 was equipped with a Crest V2 spinning disc confocal and a Prime 95B 25 mm camera (Teledyne). For confocal fluorescence imaging, illumination was supplied by a Celesta Light Engine (Lumencor) with the following excitation wavelengths, power, and emission filters: Cy5, 561 nm, 50%, Semrock 685/40m; FM4–64, 510 nm, 50%, Semrock 685/40 nm; DAPI, 408 nm, 25%, Chroma ET460/50m. Microscope 3 was equipped with an AX R point scanning confocal system with a Nikon Spatial Array Confocal detector, and 488 nm illumination was supplied by a Nikon LUA-S4 laser launch at 50% power and the following conditions: FM4–64, proprietary quad-band emission filter (677/28m); Trans, transmitted light simultaneously supplied by the 488 nm excitation light with a gain of 30 and pinhole size of 16.1.

##### Short-term sorbitol treatments of sporangia

Cells were imaged with a 40x objective (plan flour, air, NA=0.60) using the Microscope-1-Cy5 configuration. Time lapse imaging was performed on the middle plane of the cells at 5 s intervals for 8 min. with 400 ms exposure time. For validating the use of cross-sectional area to assess changes in cell size, cells were imaged with a 40x objective (plan fluor, oil, NA=1.3) using the Microscope-2-Cy5 configuration. Images were taken with 0.7 μm z-stacks through the sporangia every 30 s for 8 min. with 150 ms exposure time.

##### Membrane and cell wall staining during hyperosmotic shock in sporangia

Cells were imaged with a 40x objective (plan apo λD air, NA=0.95); the cell wall was visualized using the Microscope-2-DAPI configuration, and the cell membrane was visualized with the Microscope-2-FM4–64 configuration. Time lapse imaging was performed with 200 ms exposure of 408 nm illumination followed by 300 ms exposure of 510 nm illumination at 10 s intervals for 8 min.

##### Long-term sorbitol treatments of sporangia

Cells were imaged with the same objective and configuration as for the short-term experiments. Time lapse imaging was performed on the middle plane of the cells at 5 min. intervals for 120 min. with 400 ms exposure time. Three XY points per well were chosen for each treatment and all XY points were acquired in the same imaging acquisition using Nikon Elements v6.02.03.

##### Sorbitol and chemical inhibitors under agarose experiments

Cells were imaged with a 100x objective (plan apo λ, oil, NA=1.45) using the Microscope-1-Brightfield configuration with (Concanamycin A experiments) or without (all other experiments) DIC; the Microscope-1-TRITC configuration was also used when applicable to visualize PI staining. Timelapse images were taken with 50 ms brightfield exposure (iris open 3.3% or 5%), followed by 100 ms of 550 nm excitation when applicable, every 250 ms for 1.1 min. (Concanamycin A experiments) or 3 min. (all other experiments).

##### Under agarose membrane staining in zoospores

Cells were imaged with a 60x objective (plan apo λD, oil, NA=1.42) using the Microscope-3-Trans and Microscope-3-FM4–64 configurations. Images were taken at 5 s intervals for 2 min. using a galvano unidirectional band scanner with a dwell time of 0.4 μs.

##### Chemical inhibitors, actin, and nuclear staining

Cells were imaged at 100X (plan apo λ, oil, NA=1.45) using Microscope-1-Brightfield with DIC, Microscope-1-DAPI, and Microscope1-GFP configurations. 3D imaging was performed using 0.2 μm z-stacks through the zoospores with 100 ms brightfield and 488 nm illumination exposure, and 500 ms 402 nm illumination exposure.

##### Mucin-induced encystation experiments

Cells were imaged with a 100x objective (plan apo λ, oil, NA=1.45) using the Microscope-1-Brightfield configuration and Microscope-1-Cy5 configuration to visualize whole cells and the cell wall via Evans Blue. Timelapses were taken at 250 ms intervals for 1.5 min. using 50 ms brightfield exposure (iris open 3.3%) and 50 ms 500 nm illumination exposure every 100 frames.

#### Putative homolog identification

##### High osmolarity glycerol (HOG) pathway

Putative homologs of key components of the HOG pathway in *Bd* and other chytrids were identified by ortholog group analysis, domain analysis, and database and literature searches (Figure S2A; Data S1C). The following species were included in this analysis: *Allomyces macrogynus* ATCC38327 (*Am*; GenBank: GCA_000151295.1; WGS NCBI BioProject: PRJNA20563); *Aspergillus nidulans* FGSC A4 ^65^ (*An*; NCBI RefSeq: GCF_000011425.1); *Batrachochytrium dendrobatidis* JEL423 ^66^ (*Bd*; GenBank: GCA_000149865.1); *Candida albicans* SC5314 ^67^ (*Ca*; NCBI RefSeq: GCF_000182965.3); *Magnaporthe (Pyricularia) oryzae* 70–15 ^70^ (*Mo*; NCBI RefSeq: GCF_000002495.2); *Neurospora crassa* OR74A ^72^ (*Nc*; NCBI RefSeq: GCF_000182925.2); *Saccharomyces cerevisiae* S288C ^73^ (*Sc*; NCBI RefSeq: GCF_000146045.2); *Schizosaccharomyces pombe* 972h-^74^ (*Spo*; NCBI RefSeq: GCF_000002945.1); and *Spizellomyces punctatus* DAOM BR117 ^75^ (*Sp*; NCBI RefSeq: GCF_000182565.1). For all proteins, splice variants were not considered.

HOG pathway mitogen activated kinase (MAPK) cascade components were collected from Table S4 of a previous study, which identified these components using phylogenetic analysis.^79^ For each protein and species of interest, the reported GenInfo Identifiers (gi) were used to identify the associated NCBI/GenBank entry and accession number. In any cases where the gi linked to non RefSeq assemblies, or records that were superseded or suppressed, BLAST ^59^ was used to identify the protein in the RefSeq assembly.

Non-MAPK cascade proteins in the HOG pathway were identified by searching for putative *Am*, *Bd*, and *Sp* homologs in the OrthoMCL database ^61,80^ orthogroups that contained known *S. cerevisiae* (*Sc*) HOG pathway proteins (Data S1A). The protein domains of the resulting candidates were analyzed using InterProScan with default settings ^60^ and chytrid proteins missing one or more domains predicted to be in the *Sc* protein were removed from the dataset. If an orthogroup did not produce a candidate protein in a given species, BLASTp and/or tBLASTn (via NCBI) was used to manually identify a potential candidate using the *Sc* protein sequence as the query. For BLASTp, the nr database was used, filtering results to consider only the chytrid species of interest, a word size of three, and all other parameters set to the default settings. For tBLASTn, the search was performed on default settings against only the species of interest’s genome. The top five hits from each species were then used as the queries to do a reverse BLASTp search using the RefSeq database and filtering to consider only *Sc* (parameters as described for forward BLASTp search). Chytrid proteins for which their mutual best BLAST hit (MBBH) was the query *Sc* protein, and also contained all the expected domains from InterProScan, were considered a putative homolog. If still no clear putative homolog was found, then the protein was marked as not found.

Identification of non-MAPK cascade proteins in the HOG pathway of Dikarya (*An*, *Ca*, *Mo*, *Nc*, and *Spo*): *Spo*, the “protein with orthologs” function on pombase.org ^81^ was used to identify proteins that have a homolog in *Sc*, followed by finding each protein’s corresponding NCBI RefSeq accession number; *Ca*, each *Sc* protein was searched for on candidagenome.org, ^82^ ensuring that the *Sc* protein was under the “ortholog(s) in non-CGD species” section in the given gene page and the “external links” section was used to find the corresponding NCBI RefSeq accession; *An*, *Mo*, and *Nc* the “identify genes based on a list of IDs” option on fungidb.org ^83,84^ was used. The list of *Sc* systematic names was used as the input and a step was added to “transform [the] results into orthologs” in each of the species of interest. Entrez and Uniprot IDs for the predicted homologs were downloaded and the corresponding NCBI RefSeq accession numbers were identified. If no homolog for a protein in a Dikarya species was found through these methods, the MBBH hit approach outlined above was used, using the RefSeq database and filtering for the species of interest only for the forward search. If still no homolog for a protein was found, a literature review for the protein of interest was performed. The protein was marked as not found if none of the above methods returned a candidate.

##### Vacuolar ATPase (v-ATPase) and aquaporins

Domain analysis and BLAST searches were performed to identify putative homologs of v-ATPase subunits and aquaporins (Figure S2B; Data S1D) in the same species of interest as with the HOG pathway homolog search, with these additional species: *Acanthamoeba castellanii* str. Neff ^63^ (*Ac*; NCBI RefSeq: GCF_000313135.1); *Arabidopsis thaliana* ^64^ (*At*; NCBI RefSeq: GCF_000001735.4); *Chlamydomonas reinhardtii* ^68^ (*Cr*; NCBI RefSeq: GCF_000002595.2); *Dictyostelium discoideum* AX4 ^69^ (*Dd*; NCBI RefSeq: GCF_000004695.1); *Homo sapiens* (*Hs*; NCBI RefSeq: GCF_000001405.40); *Naegleria gruberi* ^71^ (*Ng*; NCBI RefSeq: GCF_000004985.1); *Trypanosoma brucei* 927/4 GUTat10.1 ^76^ (*Tb*; NCBI RefSeq: GCF_000002445.2); and *Trypanosoma cruzi* CL Brener ^77^ (*Tc*; NCBI RefSeq: GCF_000209065.1). Splice variants were not considered.

The v-ATPase is a multisubunit protein complex. ^48^ For *Sc*, each subunit of the v-ATPase can be uniquely identified via Interpro family or domain membership (https://www.ebi.ac.uk/interpro/; v96.0 through v100.0). The most descriptive Interpro entry accession numbers for each subunit from *Sc* were identified (Data S1B). The *c*, *c*’, and *c*” subunits are difficult to distinguish from each other, ^49^ so were combined into one category. Putative homologs were identified by inclusion in each subunit’s Interpro entry and their associated NCBI/GenBank accession numbers identified through their Uniprot entry page or BLAST via NCBI. If no candidate subunit homologs were identified in a given species, manual BLAST searches were performed as for the HOG pathway proteins. If a protein could still not be identified, it was marked as not present in the species. The c/c’/*c”* and *a* subunits from *Bd*, *Sc*, and *Dd* were aligned (Figure S3A) using TCoffee on default settings. ^62^

Putative Aquaporins were identified by inclusion in the Interpro major intrinsic protein (MIP, Interpro: IPR000425) family. ^85,86^ When available, only reviewed protein entries were counted. All proteins’ associated NCBI/GenBank accession numbers were found through their Uniprot entry page or BLAST via NCBI. Inactive accession numbers were removed from the dataset.

### QUANTIFICATION AND STATISTICAL ANALYSIS

Automated pipelines and code used in these analyses can be found on Zenodo (https://doi.org/10.5281/zenodo.16329705). Residual plots and quantile-quantile plots were used to test if the statistical approach used was appropriate for the data. All statistical values from the below analyses can be found in the main text and figure legends of this article. All graphing and statistical analyses were performed in Prism v10 (GraphPad).

#### Hyperosmotic shock of sporangia

Short-term time lapse of sporangia (Figures 1A and 1B): The frames corresponding to one minute before and one, three and five minutes after media replacement were isolated and then processed using a custom General Analysis 3 pipeline in Nikon Elements v6.02.03. Each image was adjusted to increase contrast using a rolling ball average, then thresholded and tracked to identify individual sporangia over time (example binary shown in Figure S1A). Objects with diameters less than 10 μm, along with those that touched the edges of the image, were discarded, and the area of remaining objects measured. Prior to any calculations, objects not present in all four frames as well as objects with areas below 130 μm^2^ or above 500 μm^2^ (objects representing partial or multiple adjacent cells) were removed using a custom python script. The percent change in cross-sectional area for each cell was then calculated by dividing the area of each cell in each after-treatment frame by the area of the same cell in the before-treatment frame. Statistical analysis between each treatment was performed on the average percent change in area for each of the three biological replicates using a one-way ANOVA with Tukey’s multiple comparisons. To compare measured volume to estimated volume from a cross section, and how well these metrics determine the change in cell size (Figure S1C), images were 3D thresholded using NIS element v6.02.03, 10 random cells per image were selected, and their measured volume for one minute before and five minutes after treatment were recorded. Then, images were thresholded again using the same parameters, but overlapping objects from different z-stacks were not concatenated into one 3D object, allowing for the measurement of the area of the middle cross section of the chosen cells. The area for the middle cross section for one minute before and five minutes after treatment were recorded, then the radius of the cell was estimated, which was then used to estimate volume. For both the measured and estimated volume of each cell, the percent of original volume was calculated as described, and then plotted against each other. Plasma membrane delamination (Figures 1C, 1D, and S1D): Samples were blinded and delamination was manually quantified as a clear separation between the plasma membrane and cell wall signal, or plasma membrane circles adjacent to the cell periphery. A one-way ANOVA with Tukey’s multiple comparisons on the replicate averages for each sorbitol treatment was performed. Long-term time lapse of sporangia (Figure 1E): Individual XY points were processed using the same General Analysis 3 pipeline used for the short term experiments (example cells shown in Figure S1B), objects not in all 25 time frames removed, and the same area cut-off range filter used as for the short term experiments. For all 25 frames, the percent of the original area for each cell was calculated as for the short term experiments using a custom python script.

#### Contractile vacuoles in zoospores

Sorbitol treatments under agarose (Figure 3A): Samples were blinded, five representative cells were selected per treatment condition, and image frames corresponding to the first 60 seconds of imaging were analyzed using Fiji (v1.53r).^58^ Each vacuole that disappeared was counted towards the pumping rate. The area of each shrinking vacuole was measured at its largest using the wand (tracing) tool or by freehand selection tool as was the area of each cell. The “percent cell area pumped per minute” was determined by dividing the sum of contractile vacuole areas at their largest by the cell’s area. To quantify contractile vacuole dynamics over time (Figure 2A), a representative cell from the 0 mM sorbitol condition was chosen, and every contractile vacuole was measured for each frame of the video for 60 seconds. Bafilomycin-A1 treatments under agarose (Figure 3B): contractile vacuole sizes, pumping rate, and percent of cell area pumped per minute were determined using the same methods as for the under agarose sorbitol treatments. Cell death (Figure S3D) was evaluated by positive PI staining. Cells that were clearly encysted (small and nearly perfect circles), that touched the border of the image, or were out of focus were not included in analysis. Two-tailed Student’s t-tests were performed on the measurements for multiple pumps and cell death. Concanamycin-A treatments under agarose (Figure S3F): contractile vacuole pumping rate was determined using the same methods as for the under agarose sorbitol treatments.

##### Encystation

For encystation experiments (Figure 4), time points were blinded, five randomly selected cells per replicate chosen, and the average cell wall intensity for the first frame was measured using the free-hand selection tool in Fiji to outline the cell. The pumping rate at each time point was quantified as described for the sorbitol treatments under agarose. The time between appreciable cell wall accumulation and the cessation of vacuole pumping was defined as the time difference between the first time point at which the cell’s total cell wall intensity was at least 20% brighter than background and the time point in which a cell had no pumping contractile vacuoles.

## Supplementary Material

1

2

3

4

5

6

SUPPLEMENTAL INFORMATION

Supplemental information can be found online at https://doi.org/10.1016/j.cub.2025.10.013.

## Figures and Tables

**Table:** KEY RESOURCES TABLE

REAGENT or RESOURCE	SOURCE	IDENTIFIER
Chemicals, peptides, and recombinant proteins		
Sorbitol	Sigma	Cat# S1876; CAS No. 50–70-4
Calcofluor White with Evans Blue counterstain	Sigma	Cat# 18909; CAS No. 4193–55-9 and CAS No. 314–13-6
Calcofluor White	Sigma	Cat# 910090; CAS No. 4193–55-9
Propidium iodide	Invitrogen	Cat# P3566; CAS No. 25535–16-4
FM4–64	Invitrogen	Cat# T13320; CAS No. 162112–35-8
Low melt agarose	Thermo Scientific	Cat# R0801; CAS No. 9012–36-6
Bafilomycin-A1	Cayman Chemical	Cat# 11038; CAS No. 888999–55-2
Purified mucin	Sigma	Cat# M1778; CAS No. 84082–64-4
Concanamycin-A	Tocris	Cat# 2656; CAS No. 80890–47-7
Concanavalin-A	Sigma	Cat# C2010; CAS No. 11028–71-0
Triton-X 100	Sigma	Cat# T9284; CAS No. 9036–19-5
DAPI	Life Technologies	Cat# D1306; CAS No. 28718–90-3
AlexaFlour488 phalloidin	Cell Signaling Technology	Cat# 8878S; CAS No. 17466–45-4
Deposited data		
Original analysis pipeline and custom python code	This paper	Zenodo: https://doi.org/10.5281/zenodo.16329705
Accession numbers related to osmoregulation proteins putative homolog identification	This paper	Data S1
Experimental models: Organisms/strains		
*Batrachochytrium dendrobatidis* strain JEL423	Joyce Longcore, available at CZEUM (https://czeum.herb.lsa.umich.edu/one-strain/?recordid=JEL0423 )	JEL423, or JEL0423
Software and algorithms		
NIS Elements with Advanced Research Package v6.02.03 and General Analysis 3 module	Nikon	https://www.microscope.healthcare.nikon.com/products/software/nis-elements/niselements-advanced-research
Python v3.0 or newer	Python Software Foundation	https://www.python.org/downloads/
FIJI v1.53r	Schindelin et al. ^58^	https://imagej.net/software/fiji/downloads
Basic local alignment search tool (BLAST)	Altschul et al.^59^	https://blast.ncbi.nlm.nih.gov/Blast.cgi
Interpro Database	https://www.ebi.ac.uk/interpro/	N/A
InterproScan v5.75–106.0	Jones et al. ^60^	https://www.ebi.ac.uk/interpro/search/sequence/
OrthoMCL database	Chen et al.^61^	https://orthomcl.org/orthomcl/app
TCoffee	Notredame et al.^62^	https://tcoffee.crg.eu/
UniprotKB	https://www.uniprot.org/	N/A
Other		
*Acanthamoeba castellanii* str. Neff NCBI RefSeq genome assembly	Clarke et al.^63^	NCBI RefSeq: GCF_000313135.1
*Allomyces macrogynus* ATCC38327 genome assembly (A_macrogynus_V3)	WGS project: PRJNA20563	GenBank: GCA_000151295.1
*Arabidopsis thaliana* NCBI RefSeq genome assembly	Swarbreck et al.^64^	NCBI RefSeq: GCF_000001735.4
*Aspergillus nidulans* FGSC A4 NCBI RefSeq genome assembly	Wortman et al.^65^	NCBI RefSeq: GCF_000011425.1
*Batrachochytrium dendrobatidis* JEL423 genome assembly	Farrer et al. ^66^	GenBank: GCA_000149865.1
*Candida albicans* SC5314 NCBI RefSeq genome assembly	Jones et al.^67^	NCBI RefSeq: GCF_000182965.3
*Chlamydomonas reinhardtii* NCBI RefSeq genome assembly	Merchant et al.^68^	NCBI RefSeq: GCF_000002595.2
*Dictyostelium discoideum* AX4 NCBI RefSeq genome assembly	Eichinger et al.^69^	NCBI RefSeq: GCF_000004695.1
*Homo sapiens* NCBI RefSeq genome assembly	NCBI	NCBI RefSeq: GCF_000001405.40
*Magnaporthe (Pyricularia) oryzae* 70–15 NCBI RefSeq genome assembly	Dean et al. ^70^	NCBI RefSeq: GCF_000002495.2
*Naegleria gruberi* NCBI RefSeq genome assembly	Fritz-Laylin et al. ^71^	NCBI RefSeq: GCF_000004985.1
*Neurospora crassa* OR74A NCBI RefSeq genome assembly	Galagan et al.^72^	NCBI RefSeq: GCF_000182925.2
*Saccharomyces cerevisiae* S288C NCBI RefSeq genome assembly	Engel et al.^73^	NCBI RefSeq: GCF_000146045.2
*Schizosaccharomyces pombe* 972h- NCBI RefSeq genome assembly	Wood et al.^74^	NCBI RefSeq: GCF_000002945.1
*Spizellomyces punctatus* DAOM BR117 genome assembly (S_punctatus_V1)	Russ et al. ^75^	NCBI RefSeq: GCF_000182565.1
*Trypanosoma brucei* 927/4 GUTat10.1 NCBI RefSeq genome assembly	Berriman et al.^76^	NCBI RefSeq: GCF_000002445.2
*Trypanosoma cruzi* CL Brener NCBI RefSeq genome assembly	El-Sayed et al. ^77^	NCBI RefSeq: GCF_000209065.1
Dynamic cell confiner	4D Cell	https://www.4dcell.com/smartconfinement/dynamic-confiner

## Highlights

- Chytrid fungi regulate pressure differently in cell types with and without cell walls
- Walled chytrid sporangia maintain high turgor pressure, similar to other fungi
- Non-walled zoospores use contractile vacuoles to expel water, reducing pressure
- Contractile vacuole activity tapers as the wall forms during sporangial development
