# Supplementary material for: Frog-killing chytrid fungus deploys different strategies to regulate intracellular pressure in developmental states that have or lack a cell wall
Source: Curr Biol. Author manuscript; Available in PMC 2026 Jan 20. (PMC12818911; doi:10.1016/j.cub.2025.10.013)
Supplement: 6 [file NIHMS2116685-supplement-6.pdf]

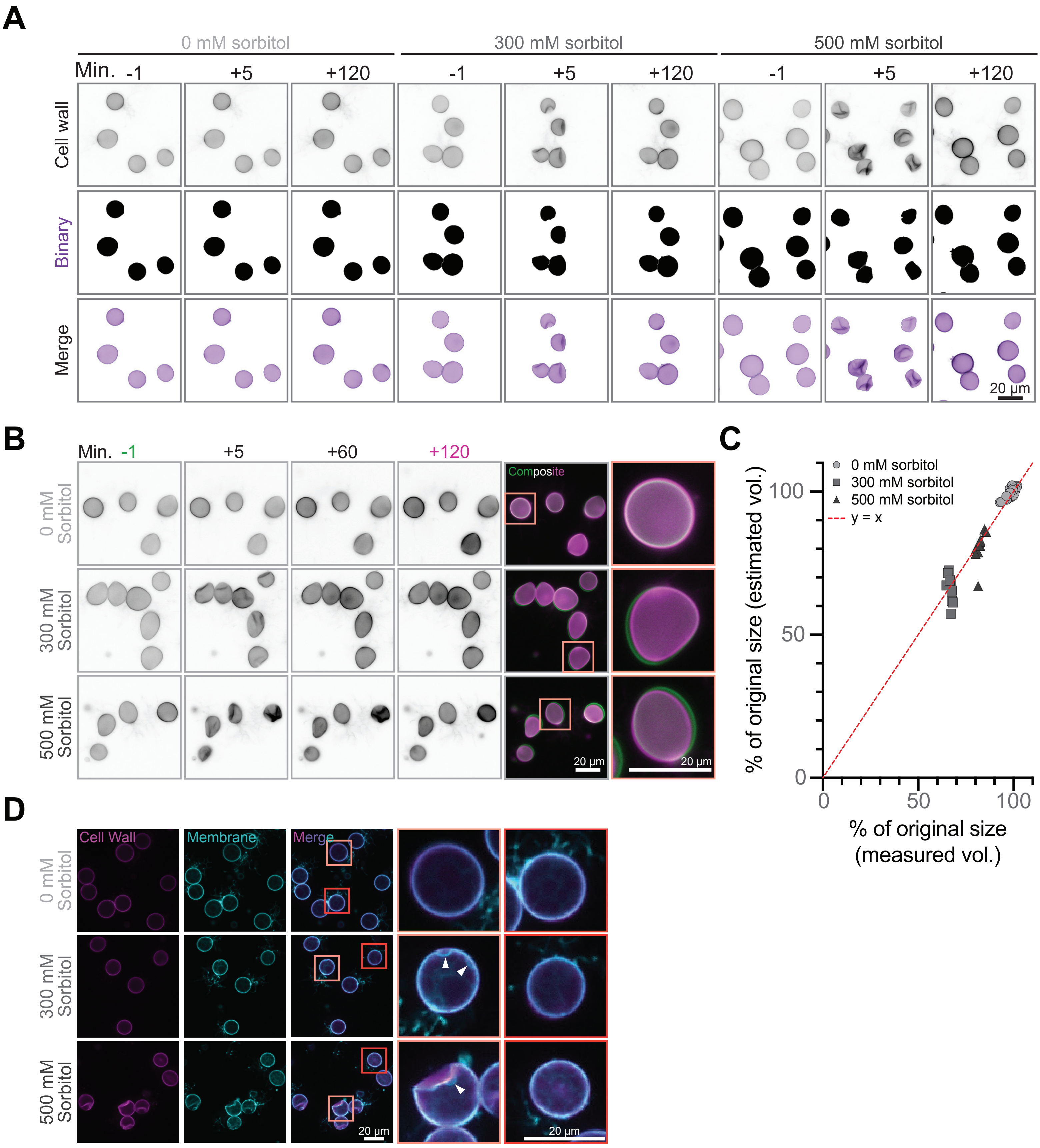
A
0 mM sorbitol
300 mM sorbitol
500 mM sorbitol
Min. -1
+5
+120
Cell wall
Binary
Merge
20 µm
B
Min. -1
+5
+60
+120
Composite
0 mM Sorbitol
300 mM Sorbitol
500 mM Sorbitol
20 µm
C
0 mM sorbitol
300 mM sorbitol
500 mM sorbitol
y = x
% of original size (estimated vol.)
% of original size (measured vol.)
0
50
100
D
Cell Wall
Membrane
Merge
0 mM Sorbitol
300 mM Sorbitol
500 mM Sorbitol
20 µm

Figure S1. Analysis of *Bd* sporangia responding to hyperosmotic shock, related to Figure 1. **(A)** Representative images of *Bd* sporangia cell walls stained with Evans Blue (top) one minute before (-1) and five (+5) and 120 (+120) minutes after treatment with media supplemented with the indicated sorbitol concentration. All cell wall images are adjusted to the same brightness and contrast. Cells were segmented using the cell wall signal in NIS elements (v6.02.03), resulting in a binary layer (purple) that encompasses the cell body. **(B)** Representative images of *Bd* sporangia cell walls stained with Evans Blue one minute before (-1) and five (+5), 60 (+60) and 120 (+120) minutes after treatment with media supplemented with the indicated concentration of sorbitol. Composite images show cells one minute before (green) and 120 minutes after (magenta) sorbitol treatment. All images are adjusted to the same brightness and contrast. **(C)** The percent of pre-treatment volume five minutes after the given sorbitol treatment based on measured volume from a 3D stack compared to estimated volume from a cross-sectional area for the same cell. 0 mM, n = 40 cells; 300 mM, n = 10; 500 mM, n = 10). **(D)** Example images of *Bd* sporangia stained for the cell wall (magenta) and membrane (cyan) after five minutes of treatment with media supplemented with the given sorbitol concentration. Merged images show the overlay of membrane and cell wall. For the 300 and 500 mM sorbitol treatments, insets give one example of a cell with delamination and another example of a cell without delamination from the same field of view. White arrowheads indicate membrane delaminations. All images for each stain are adjusted to the same brightness and contrast.

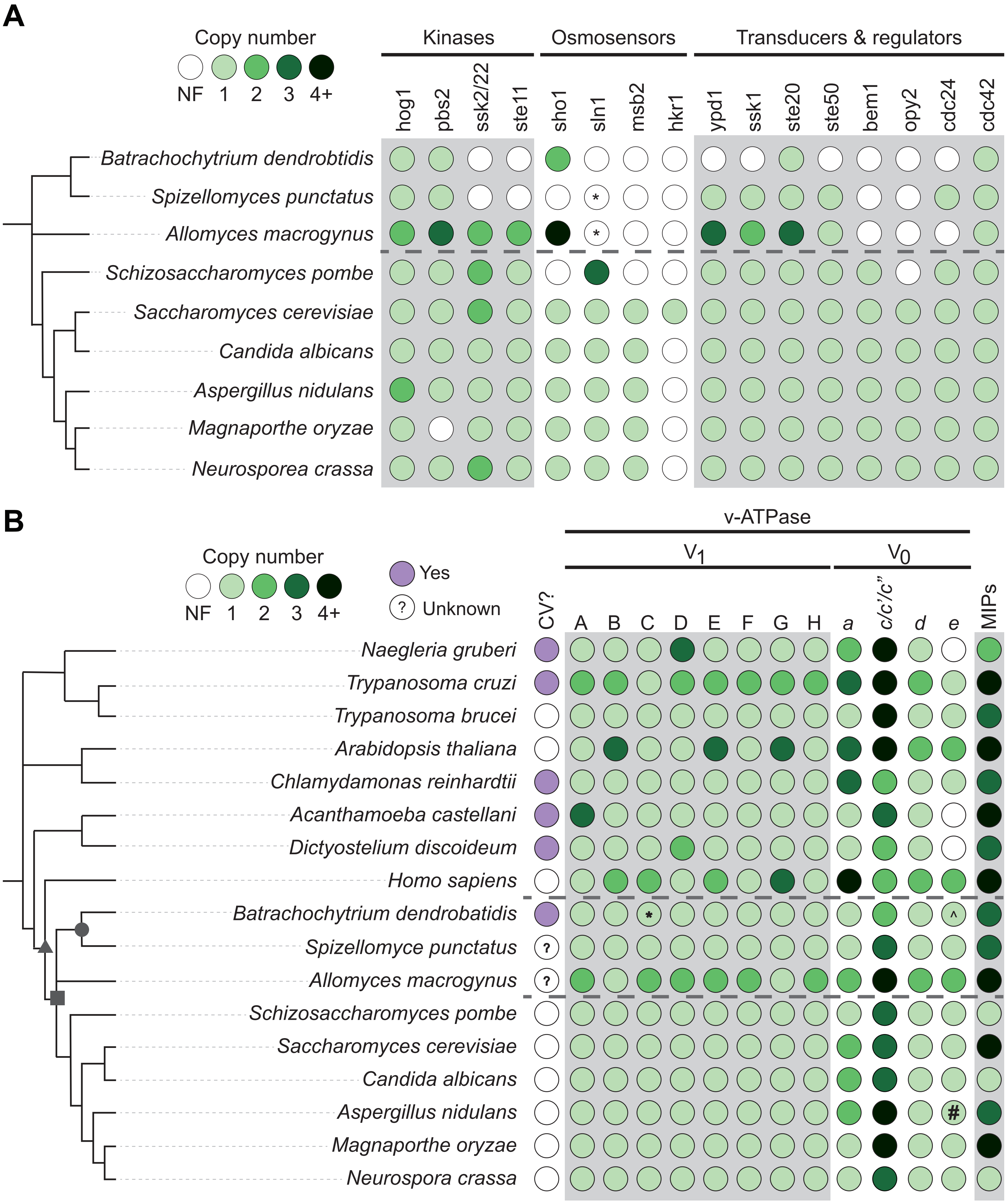
A
Copy number
NF 1 2 3 4+
Kinases
Osmosensors
Transducers & regulators
hog1
pbs2
ssk2/22
ste11
sho1
sln1
msb2
hkr1
ypd1
ssk1
ste20
ste50
bem1
opy2
cdc24
cdc42
Batrachochytrium dendrobtidis
Spizellomyces punctatus
Allomyces macrogynus
Schizosaccharomyces pombe
Saccharomyces cerevisiae
Candida albicans
Aspergillus nidulans
Magnaporthe oryzae
Neurosporea crassa
B
v-ATPase
Copy number
NF 1 2 3 4+
Yes
Unknown
CV?
A
B
C
D
E
F
G
H
a
c/c'/c"
d
e
MIPs
Naegleria gruberi
Trypanosoma cruzi
Trypanosoma brucei
Arabidopsis thaliana
Chlamydamonas reinhardtii
Acanthamoeba castellani
Dictyostelium discoideum
Homo sapiens
Batrachochytrium dendrobatidis
Spizellomyce punctatus
Allomyces macrogynus
Schizosaccharomyces pombe
Saccharomyces cerevisiae
Candida albicans
Aspergillus nidulans
Magnaporthe oryzae
Neurospora crassa

Figure S2. *Bd* and other chytrids have putative homologs of several key components involved in osmoregulation, related to Figures 1 and 2.

(A) The distribution of proteins known to function in the high osmolarity glycerol pathway (HOG) across fungal taxa. The HOG pathway is the primary pathway used by budding yeast to regulate turgor pressure and has three main classes of proteins: 1) a mitogen activated kinase (MAPK) cascade ending with the MAPK Hog1; 2) osmo-sensors to sense changes in external osmolarity; and 3) transducers and regulators that connect the MAPKs and osmosensors. White-filled circles indicate that homologs are not found (NF), color-filled circles indicate the detection of one or more homologs. Kinase copy numbers were obtained from[S1]. Dark dashed line separates chytrid species (above) from Dikaryotic species (below). *: There is no clear homolog for sln1, but there are putative histidine kinases with predicted transmembrane domains in the given species' genomes. Transmembrane domains are a hallmark of sln1-related histidine kinases[S2, S3] . **(B)**The distribution of major intrinsic family proteins (MIPs), subunits of the vacuolar-ATPase (v-ATPase), and presence of contractile vacuoles (CV) across taxa. Aquaporins are part of the MIP family of proteins. The v-ATPase is made up of two complexes (V1 and V0), each comprising several subunits[48]. White-filled circles indicate that homologs are not found (NF), color-filled circles indicate the detection of one or more homologs. Purple circles indicate the presence of documented CVs in the given organism. *: Homolog is predicted to be in the ubiquitin activating enzyme family (IPR018075), but this likely represents two separate genes erroneously annotated as one. ^: Homolog only identifiable when using the Manduca sexta protein (NCBI RefSeq XP_037299296.1) as a query for BLASTp or tBLASTn. #: Homolog is not annotated in the reference genome, and is only identifiable when using the Sc protein as a query for tBLASTn. Symbols on the tree represent opisthokonts (triangle), fungi (square), and Chytridiomycota (circle). Dark dashed lines surround chytrid species.

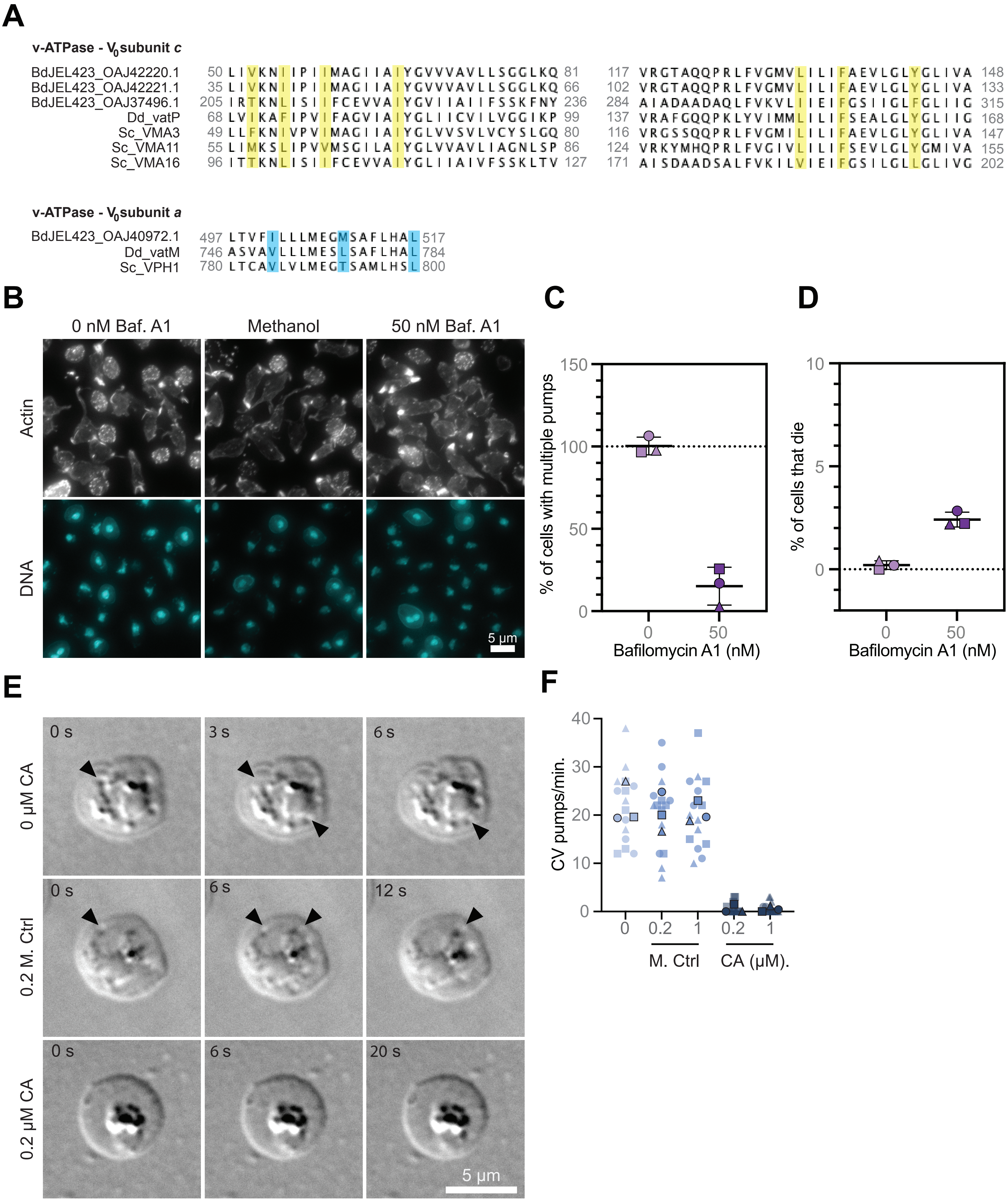

A
v-ATPase - V0 subunit c
BdJEL423_OAJ42220.1 50 LIVKNIIPIIMAGIIAIYGVVVAVLLSGGLKQ 81 117 VRGTAQQPRLFVGMVLILIFAEVLGLYGLIVA 148
BdJEL423_OAJ42221.1 35 LIVKNIIPIIMAGIIAIYGVVVAVLLSGGLKQ 66 102 VRGTAQQPRLFVGMVLILIFAEVLGLYGLIVA 133
BdJEL423_OAJ37496.1 205 IRTKNLISIIFCEVVAIYGVIIAIIFSSKFNY 236 284 AIADAADAQLFVKVLIIEIFGSIIGLFGLIIG 315
Dd_vatP 68 LVIKAFIPVIFAGVIAIYGLIICVILVGGIKP 99 137 VRAFGQQPKLYVIMMLILIFSEALGLYGLIIG 168
Sc_VMA3 49 LLFKNIVPVIMAGIIAIYGLVVSVLVCYSLGQ 80 116 VRGSSQQPRLFVGMILILIFAEVLGLYGLIVA 147
Sc_VMA11 55 LIMKSLIPVVMSGILAIYGLVVAVLIAGNLSP 86 124 VRKYMHQPRLFVGIVLILIFSEVLGLYGMIVA 155
Sc_VMA16 96 ITTKNLISIIFCEVVAIYGLIIAIVFSSKLTV 127 171 AISDAADSALFVKILVIEIFGSILGLLGLIVG 202
v-ATPase - V0 subunit a
BdJEL423_OAJ40972.1 497 LTVFILLLMEGMSAFLHAL 517
Dd_vatM 746 ASVAVLLLMESLSAFLHAL 784
Sc_VPH1 780 LTCAVLVLMEGTSAMLHSL 800
B
0 nM Baf. A1
Methanol
50 nM Baf. A1
Actin
DNA
5 µm
C
% of cells with multiple pumps
150
100
50
0
0
50
Bafilomycin A1 (nM)
D
% of cells that die
10
5
0
0
50
Bafilomycin A1 (nM)
E
0 µM CA
0 s
3 s
6 s
0.2 M. Ctrl
0 s
6 s
12 s
0.2 µM CA
0 s
6 s
20 s
5 µm
F
CV pumps/min.
40
30
20
10
0
0
0.2
1
0.2
1
M. Ctrl
CA (µM).

Figure S3. *Bd* is susceptible to the vacuolar ATPase inhibitor Bafilomycin A1, related to Figure 3. **(A)** TCoffee alignment of known and putative homologs for the indicated vacuolar ATPase subunits in the given species. Residues that form the Bafilomycin A1 binding site (yellow) or that are disrupted by bafilomycin A1 binding (cyan) are highlighted. *Bd*, *Batrachochytrium dendrobatidis strain* JEL423; *Dd*, *Dictyostelium discoideum* AX4; *Sc*, *Saccharomyces cerevisiae* 288C. **(B)** *Bd* zoospores were treated with or without 50 nM Bafilomycin A1, or equal amount of methanol as a vehicle control, and then fixed and stained for actin using phalloidin (grey) and the nucleus using DAPI (cyan). All images for each stain are adjusted to the same brightness and contrast. **(C)** Quantification of the percent of *Bd* zoospores that exhibit organelles undergoing multiple growing and shrinking cycles over a three minute period under agarose after 35 minutes of treatment with 0 or 50 nM bafilomycin A1. Three independent biological replicates were performed, each represented by a shape. Mean and standard deviation of the three biological replicates are indicated by black lines. Two-tailed Student's t-test: $p = 0.0007$. **(D)** Quantification of the percent of *Bd* zoospores that die over a three minute period under agarose treated with 0 or 50 nM Bafilomycin A1. Calculated by taking the difference between the percent of propidium iodide positive cells between the last and first frames of the time lapse. Three independent biological replicates were performed, each represented by a shape. Mean and standard deviation of the three biological replicates are indicated by black lines. Two-tailed Student's t-test: $p = 0.0008$. **(E)** Representative timelapse images of *Bd* zoospores under agarose treated with or without 0.2 μM of the vacuolar ATPase inhibitor Concanamycin A (CA), or equal volume of methanol (M. Ctrl), for 35 minutes. Images are on an inverted LUT. **(F)** quantification of vacuole pumping rate in five random cells per treatment with or without Concanamycin A (CA), or equal volume of methanol (M. Ctrl) for 35 minutes.

Supplemental References

S1. Xu, C., Liu, R., Zhang, Q., Chen, X., Qian, Y., and Fang, W. (2017). The Diversification of Evolutionarily Conserved MAPK Cascades Correlates with the Evolution of Fungal Species and Development of Lifestyles. Genome Biol Evol *9*, 311–322.

S2. Catlett, N.L., Yoder, O.C., and Turgeon, B.G. (2003). Whole-genome analysis of two-component signal transduction genes in fungal pathogens. Eukaryot Cell *2*, 1151–1161.

S3. Defosse, T.A., Sharma, A., Mondal, A.K., Dugé de Bernonville, T., Latgé, J.-P., Calderone, R., Giglioli-Guivarc'h, N., Courdavault, V., Clastre, M., and Papon, N. (2015). Hybrid histidine kinases in pathogenic fungi. Mol Microbiol *95*, 914–924.

S4. Vasanthakumar, T., and Rubinstein, J.L. (2020). Structure and Roles of V-type ATPases. Trends Biochem Sci *45*, 295–307.
